# Novel mitochondrial mechanisms of cognitive regulation in subjects with cognitive impairments

**DOI:** 10.1192/j.eurpsy.2023.1274

**Published:** 2023-07-19

**Authors:** B. Bigio, R. Lima-Filho, O. Barnhill, F. Sudo, C. Drummond, N. Assunção, B. Vanderborght, F. Tovar-Moll, P. Mattos, S. Ferreira, F. De Felice, M. Lourenco, C. Nasca

**Affiliations:** 1 Department of Psychiatry, New York University Grossman School of Medicine, New York, United States; 2 Institute of Medical Biochemistry Leopoldo de Meis, Rio de Janeiro, Brazil; 3 Rockefeller University, New York, United States; 4 D’Or Institute for Research and Education (IDOR), Rio de Janeiro, Brazil; 5 Queen’s University, Kingston, Canada; 6 Department of Neuroscience and Physiology, New York University Grossman School of Medicine, New York, United States

## Abstract

**Introduction:**

Prior mechanistic studies in rodents showed decreased levels of the pivotal mitochondrial metabolite acetyl-L-carnitine (LAC) in relation to cognitive deficits and depressive-like behavior (Neuron 2017, 10.1016/j.neuron.2017.09.020, PNAS 2013, 10.1073/pnas.1216100110), providing the basis for the current translational study.

**Objectives:**

The main objective of this work was to ascertain the role of this specific mitochondrial signaling pathway in subjects with cognitive impairments (CI), and potential sex differences in these mechanisms.

**Methods:**

We used computational approaches, ultraperformance liquid chromatography–tandem mass spectrometry (UPLC-MS/MS) and available plasma samples from a well-characterized cohort of 71 subjects, including subjects with CI and age- and sex-matched cognitively healthy controls (HC).

**Results:**

Our newest findings showed decreased levels of LAC in subjects with CI as compared to age- and sex-matched HC. We also found important sex differences in carnitine levels in relation to cognitive function as assessed by using the Mini Mental Status Exam (MMSE). Specifically, the degree of carnitine deficiency reflected the severity of cognitive dysfunction in a sex-specific manner. Using computational approaches, we found that the integration of these mitochondrial measures with canonical biomarkers improves diagnostic accuracy.

**Conclusions:**

The current findings of sex differences in carnitine deficiency in subjects with CI suggest a possible sex-specific mitochondrial phenotype of vulnerability to cognitive dysfunction, and point to LAC-related mitochondrial metabolism as a new signaling pathway of cognitive regulation.

**Disclosure of Interest:**

None Declared

